# Effects of a single bout of exercise on human hemocytes and serum interleukin 3, erythropoietin, and soluble transferrin receptor in a hot and humid environment

**DOI:** 10.7717/peerj.18603

**Published:** 2024-11-29

**Authors:** Yuhu Lv, Lin Cheng, Xiqian Zhang, Fenglin Peng, Yu Yuan, Xiquan Weng, Wen-Tao Lin

**Affiliations:** 1 College of Physical Education, Guangdong University of Education, GuangZhou, Guangdong, China; 2 Research Center for Adolescent Sports and Health Promotion of Guangdong Province, GuangZhou, Guangdong, China; 3 College of Physical Education and Health, Guangxi Normal University, Guilin, Guangxi Zhuang Autonomous Region, China; 4 Guangzhou Sport University, Guangzhou, Gaungdong, China; 5 College of Sports Science, Zhuhai College of Science and Technology, Zhuhai, Guangdong, China

**Keywords:** Single bout of exercise, Hot and humid environment, Intensity, Hemocytes, Serum hematopoietic factors

## Abstract

**Background:**

Exercise in humid and hot environments (HHEs) may result in decreased perception, motor performance, and memory owing to endogenous heat production and exogenous load. However, whether a single bout of exercise (SBOE) intensity affects the magnitude of changes in the levels of hemocytes remains controversial. In this article, we aimed to investigate the effects of a SBOE of varying intensities on blood cells in HHE.

**Methods:**

Thirty-two volunteers were randomly divided into a quiet control group (QC), 55% VO_2_max intensity exercise group (HHE55%), 70% VO_2_max intensity exercise group (HHE70%), and 85% VO_2_max intensity exercise group (HHE85%). The participants in the exercise groups were assigned to perform an SBOE on the treadmill under HHE conditions for 30 min, whereas participants in the QC remained still under HHE conditions for 30 min (temperature: 28–32 °C, relative humidity: 85–95%).

**Results:**

The net body mass (NBM), perfusion index (PI), mean corpuscular volume (MCV), platelet (PLT), and plateletcrit (PCT) values were affected significantly by the exercise intensity (*P* < 0.01) the hemoglobin (HGB) and neutrophil count (NE) were affected significantly by exercise intensity (*P* < 0.05). After an SBOE, compared with that before exercise, the sublingual temperature (ST) of all groups, the NBM and MCV of all exercise groups, the PI of the HHE55% and HHE70% groups, the HGB, hematocrit (HCT), and NE of the HHE70% group, the red blood cell count (RBC), PLT, and PCT of the HHE70% and HHE85% groups, and the white blood cell count (WBC) of HHE85% changed very significantly (*P* < 0.01). The PCT of QC, blood oxygen saturation (SaO_2_), and soluble transferrin receptor (sTfR) levels in the HHE55% group, the lymphocyte count (LY) in the HHE70% group, and the HGB and HCT in the HHE85% group changed significantly (*P* < 0.05).

**Conclusion:**

Low- and moderate-intensity SBOE in HHE could increase the serum EPO and serum sTfR levels and decrease the serum IL-3 levels. Conversely, a high-intensity load could increase the risk of inflammation. Therefore, low-intensity exercise may be more appropriate for an SBOE in HHE.

## Introduction

Workers, firefighters, athletes, military personnel, and people living in specialized regions frequently face hot and humid conditions (Wijekulasuriya et al., 2022; de Korte et al., 2023; Chan et al., 2016; Wright et al., 2013; Ashworth, Cotter & Kilding, 2021; Choudhary & Udayraj, 2023). When the relative humidity (RH) reaches 70%, the body becomes more prone to fatigue (Tsutsumi et al., 2007). Humans in hot and humid environments (HHEs) are exposed to health risks and thermal discomfort that severely affect their physical, physiological, and mental workload (Golbabaei et al., 2022). Heat stress may lead to perceptual, motor, and memory loss owing to endogenous heat production and exogenous loading, which consequently reduces the quality of work, training, and exercise performance (de Korte et al., 2023; Lee et al., 2015; Nybo, 2008; De Jonge et al., 2012; Than Tran et al., 2015; Randall, Ross & Maxwell, 2015; Cleary, Toy & Lopez, 2014). Heat and humidity can also cause sleep deprivation, thus leading to liver damage and fatigue, which affects the ability of individuals to exercise (Li et al., 2014). To improve the adaptability of different populations in HHEs, researchers have conducted studies on multiple strategies, including but not limited to the use of fans (Rubin, 2019), determining the appropriate time for acclimatization (Neal et al., 2016; Garrett, Rehrer & Patterson, 2011), designing cooling apparel (McFarlin, Henning & Venable, 2017; McFarlin et al., 2016), exploring the mechanisms underlying heat stress generation (Crandall & Gonzalez-Alonso, 2010; Poirier et al., 2013), and determining the influence of thermal environment and air quality on thermal comfort (Yu, Matzarakis & Lin, 2020). A study has shown that even short-term heat acclimatization for 4 days facilitates effective perceptual adaptations without compromising immune status before an ultra-endurance race under heat stress (Willmott et al., 2017).

Appropriate exercise has various functions, including but not limited to memory enhancement, cognitive improvement, and inflammation reduction (De Miguel et al., 2021). Exercise may also have positive effects on vulnerable patient groups. A study showed that breast cancer treatment can lead to prolonged immunosuppression that leaves patients vulnerable to infection. However, exercise may be a strategy for at-risk groups, such as patients with cancer, to improve resistance to infectious disease (Bartlett et al., 2021). A single bout of exercise (SBOE) can affect blood circulation by stimulating the movement of hematopoietic bone marrow stem cells and aging immune cells from peripheral tissues into the circulatory system and stimulating immune cells both during effort and recovery (Sellami et al., 2018; Navalta et al., 2010; Simpson et al., 2010; Sardeli, Mori & Lord, 2022; Mathot et al., 2021; Dinh et al., 2017). Acute exercise can cause stress and inflammation, increase red blood cell volume and platelet compensation, affect the function of circulating hematopoietic progenitor cells, and alter the classification of white blood cells (Stelzer et al., 2015). The “open window” theory suggests that the immune system is temporarily suppressed following an acute bout of endurance exercise (Kakanis et al., 2010; Peake et al., 2017). One study showed that an SBOE drastically augments the number of cytomegalovirus and Epstein-Barr virus-specific T-cells manufactured over an 8-day period (Spielmann et al., 2016), whereas another study showed that NK cell activity is enhanced during moderate as well as severe acute exercise, whereas immunodepression is observed after severe exercise (Pedersen & Ullum, 1994). These findings suggest that lymphocyte redeployment after acute exercise may be an evolutionary conservative immune mechanism that improves our ability to resist infection. However, whether the intensity of exercise during acute exercise affects the magnitude of changes in immune cell levels remains controversial (Dinh et al., 2017; Timmerman et al., 2008). Only a limited number of studies have been conducted on the outcomes of an SBOE of different intensities under HHEs. In this study, we hypothesized that the blood cell count and levels of interleukin 3 (IL-3), erythropoietin (EPO), and soluble transferrin receptor (sTfR) in participants would alter in response to an SBOE under HHE conditions and would be affected by the exercise intensity. We determined the net body mass (NBM), sublingual temperature (ST), blood oxygen saturation (SaO_2_), perfusion index (PI), blood cell count, and hematopoietic factors of participants pre- and post-exercise and explored the effects of an SBOE in an HHE on the body. Our results preliminarily verified that the exercise intensity of SBOE can affect human blood cells, hematopoietic factors, and immune functions, which may provide a reference for further research.

## Materials and methods

### Participants

Thirty-two young men were randomly divided into a quiet control group (QC), 55% VO_2_max intensity exercise group (HHE55%), 70% VO_2_max intensity exercise group (HHE70%), and 85% VO_2_max intensity exercise group (HHE85%), with eight participants in each group. Participants in the exercise groups were assigned to perform an SBOE in an HHE for 30 min, whereas participants in the QC remained still in an HHE for 30 min (temperature: 28–32 °C, relative humidity: 85–95%). The participants included in the study had no history of hematopoietic, liver, kidney, and endocrine diseases and passed an exercise health screening. Participants took a regular diet and did not consume supplements during the study. All participants were volunteers who signed an informed consent form before the commencement of the experiment. The human study protocols were approved by the Guangzhou Sport University Human Ethics Committee (No. 2018LCLL-10). Baseline participant characteristics are shown in Table 1.

**Table 1:** Baseline participant characteristics.

	*N*	Age, years	Height, cm	Net body mass, kg	VO_2_max, mL/kg/min
QC	8	21 ± 1	173 ± 5	64.93 ± 4.79	48.70 ± 5.16
HHE55%	8	21 ± 1	172 ± 8	63.38 ± 4.57	53.44 ± 4.46
HHE70%	8	21 ± 1	175 ± 4	66.50 ± 4.57	51.34 ± 5.26
HHE85%	8	21 ± 1	175 ± 4	66.63 ± 5.34	48.33 ± 5.28

### Exercise training program

We used air conditioning (GREE, KFR-35GW/(35556) FNDe-3) and humidifiers (Midea, S35U-L, and S20U-M) to create an HHE (approximate temperature of 28 to 32 °C and approximate humidity of 85% to 95%) in a chamber. Participants from the HHE55%, HHE70%, and HHE85% groups were assigned to perform an SBOE on the treadmill in the artificial HHE for 30 min, whereas participants from the QC group remained still in the HHE for 30 min. All participants were free to drink water (*i.e*., there was no restriction on the amount of water intake) during the test.

### Measurements

In the morning before exercise and the morning after exercise, participants entered the laboratory and sat quietly for 10 min, and NBM, ST, SaO_2_, and PI were measured. Following this, 2 mL of EDTA-k_2_ anticoagulant and 5 mL of non-anticoagulant from venous blood were collected from the elbow area for routine blood examination and serum separation, respectively. Immediately after the mixing of the 2 mL of EDTA-k_2_ anticoagulant of venous blood was completed using an oscillator, a routine blood test was performed using the XS-800i Sysmex (Sysmex Co., Ltd., Kobe, Japan). The red blood cell count (RBC), hemoglobin (HGB), hematocrit (HCT), mean corpuscular volume (MCV), platelets (PLT), plateletcrit (PCT), mean platelet volume (MPV), white blood cell count (WBC), lymphocyte count (LY), neutrophil count (NE), monocyte count (MO), eosinophil count (EO), and basophil count (BA) were determined. SaO_2_ and PI were measured using a Prince-100H pulse oxygen saturation meter (Heal Force Co., Ltd., Shanghai, China). After 5 mL (non-anticoagulant) of venous blood was collected, centrifugation was performed immediately at 3,000 rpm for 10 min in a bench-top centrifuge. The serum was added to a tube and stored in the refrigerator at −20 °C for testing. Enzyme-linked immunosorbent assay (ELISA) was performed to measure serum hematopoietic factors using a TECAN Infinite M200PRO multifunctional microplate reader (Männedorf, Switzerland). All serum hematopoietic factors were measured in accordance with the manufacturer’s instructions. First, blank control, standard, and test samples were added to the ELISA plate using a single-channel pipette, and the samples were added within 10 min. In addition, all tests were performed using the same batch of ELISA reagent under the same tester to reduce intra- and inter-assay variations. Human serum IL-3 levels were measured using an IL-3 ELISA Kit (Blue Gene Biotech Co., Ltd., Shanghai, China), with the approximate standard range being 0 to 1,000 pg/mL. Human serum EPO was measured using the EPO ELISA Kit (Blue Gene Biotech Co., Ltd., Shanghai, China), the approximate standard range and sensitivity were 0 to 250 and less than 1.6 mIU/mL, respectively. Human serum sTfR was assayed using an sTfR ELISA Kit (Blue Gene Biotech Co., Ltd., Shanghai, China). The assay range was between 0 and 100 ng/mL, and the sensitivity in this assay was less than 0.39 ng/mL.

### Statistical analysis

The distribution normality of the variables was determined using a 1-sample Kolmogorov–Smirnov test and the homogeneity of variances was determined using Levene’s test in IBM SPSS Statistics 26 (IBM, Armonk, NY, USA). Normally distributed variables are presented as mean ± standard deviation (SD) and 95% confidence interval for the mean. A single-sample *t*-test was used to determine baseline participant characteristics and independent-sample *t*-test baseline participant characteristics between different groups. A paired sample *t*-test was used to analyze the pre-exercise and post-exercise indicators in the same group. One-way ANOVA was used to analyze comparisons among groups. The Bonferroni method was used to analyze the data variance homogeneity without significance. Other data with significance were analyzed using the Kruskal–Wallis test. An independent-sample *t*-test was used to analyze data that had been analyzed for significance using the Kruskal–Wallis test. Statistical significance was set at *P* < 0.05.

## Results

### Effects of SBOE on NBM, ST, SaO_2_, and PI

The results of one-way ANOVA showed that the NBM (*P* < 0.01) and PI (*P* < 0.01) were affected significantly by the exercise intensity, whereas ST (*P* > 0.05) was not affected. Results of the Kruskal–Wallis test showed that SaO_2_ (*P* > 0.05) was not affected by exercise intensity. Bonferroni’s *post hoc* comparison showed that the NBM (HHE55%, *P* < 0.01; HHE70%, *P* < 0.05; HHE85%, *P* < 0.01) and PI (HHE55%, *P* < 0.01; HHE70%, *P* < 0.01; HHE85%, *P* < 0.05) values in the test groups were significantly different from those in the QC group. However, the NBM and PI values from the HHE55%, HHE70%, and HHE85% groups showed no significant differences. The results of a paired sample *t*-test showed that, compared with the pre-exercise values, the NBM in the QC, HHE55%, HHE70%, and HHE85% groups decreased by 0.07 kg, 0.94 kg (*P* < 0.01), 0.88 kg (*P* < 0.01), and 1.07 kg (*P* < 0.01), respectively, after an SBOE in an HHE. The ST values in the QC, HHE55%, HHE70%, and HHE85% groups increased by 0.52 °C (*P* < 0.01), 0.49 °C (*P* < 0.01), 0.89 °C (*P* < 0.01), and 0.92 °C (*P* < 0.01), respectively. The SaO_2_ in the QC group increased by 0.38%, whereas the values in the HHE55%, HHE70%, and HHE85% groups decreased by 0.62% (*P* < 0.05), 0.13%, and 0.50%, respectively. The PI in the QC, HHE55%, HHE70%, and HHE85% groups increased by 0.05%, 6.56% (*P* < 0.01), 5.23% (*P* < 0.01), and 3.72%, respectively. These data are presented in Table 2.

**Table 2:** Values of NBM, ST, SaO_2_, and PI pre- and post-exercise.

	Intervention	QC (*N* = 8)	HHE55 (*N* = 8)	HHE70% (*N* = 8)	HHE85% (*N* = 8)
NBM, kg	Pre-Ex	64.88 ± 4.74	63.38 ± 4.52	66.19 ± 4.34	66.63 ± 5.59
	Post-Ex	64.81 ± 4.72	62.44 ± 4.47^b^^,^ ^d^	65.31 ± 4.04^b^^,^ ^c^	65.56 ± 5.45^b^^,^ ^d^
ST, °C	Pre-Ex	36.43 ± 0.30	36.67 ± 0.32	36.90 ± 0.39	36.81 ± 0.35
	Post-Ex	36.95 ± 0.08^b^	37.16 ± 0.30^b^	37.79 ± 0.51^b^	37.73 ± 0.64^b^
SaO_2_, %	Pre-Ex	98.50 ± 1.41	99.00 ± 0.00	97.88 ± 1.36	98.38 ± 1.41
	Post-Ex	98.88 ± 0.35	98.38 ± 0.52^a^	97.75 ± 1.58	97.88 ± 1.81
PI, %	Pre-Ex	1.73 ± 0.53	1.60 ± 0.56	3.56 ± 1.46	2.26 ± 1.57
	Post-Ex	1.78 ± 0.51	8.16 ± 2.94^b^^,^ ^d^	8.79 ± 3.21^b^^,^ ^d^	5.98 ± 5.02^c^

**Notes:**

Data are presented as mean ± standard deviation (SD).

Abbreviations: Pre-Ex, pre-exercise; Post-Ex, post-exercise; NBM, net body mass; ST, sublingual temperature; SaO_2_, blood oxygen saturation; PI, perfusion index.

a *P* < 0.05.

b *P* < 0.01, compared with Pre-Ex.

c *P* < 0.05.

d *P* < 0.01, compared with QC.

### Effects of SBOE on blood cells

One-way ANOVA showed that MCV (*P* < 0.01), PLT (*P* < 0.01), and PCT (*P* < 0.01) were affected significantly by exercise intensity, whereas the RBC, WBC, LY, MO, and EO were unaffected (*P* > 0.05). Results of the Kruskal–Wallis test showed that the HGB and NE were affected by the exercise intensity (*P* < 0.05), whereas the HCT, MPV, and BA values were unaffected by the exercise intensity (*P* > 0.05). Bonferroni’s *post hoc* comparison showed that the MCV values in the HHE55% (*P* < 0.01), HHE70% (*P* < 0.01), and HHE85% (*P* < 0.05) groups were significantly different from those in the QC groups. However, the values in the other groups did not show significant differences. The PLT (HHE70%, *P* < 0.01; HHE85%, *P* < 0.01) in two test groups was significantly different from that in the QC group, but there were no significant differences between the values in the QC group and the two remaining groups. The PCT values (HHE70%, *P* < 0.05; HHE85%, *P* < 0.01) in two test groups were significantly different from that in the QC group. The PCT value in the HHE55% (HHE70%, *P* < 0.05; HHE85%, *P* < 0.05) was significantly different from the values in two test groups. However, no significant difference was observed between the values in the QC and HHE55% groups. The independent-sample *t*-test showed that the HGB values (HHE70%, *P* < 0.01; HHE85%, *P* < 0.05) in two test groups were significantly different from that in the QC group, but there were no significant differences between the values in the two remaining groups. NE values in the remaining two groups showed no significant differences. The paired sample *t*-test showed that, compared with the pre-exercise values, after an SBOE in an HHE, the RBC in the QC, HHE55%, HHE70%, and HHE85% groups increased by 0.04 × 10^12^/L, 0.16 × 10^12^/L, 0.25 × 10^12^/L, and 0.14 × 10^12^/L, respectively. The changes in the HHE70% and HHE85% group were significant (*P* < 0.01). The HGB values decreased by 0.87 g/L in the QC group, whereas the HGB values in the HHE55%, HHE70%, and HHE85% groups increased by 5.25 g/L, 7.13 g/L, and 3.50 g/L, respectively. The changes in the HHE70% (*P* < 0.01) and HHE85% (*P* < 0.05) groups were significant. The HCT in the QC, HHE55%, HHE70%, and HHE85% groups increased by 0.36%, 0.62%, 1.67%, and 0.80%, respectively. The values in the HHE70% (*P* < 0.01) and HHE85% (*P* < 0.05) groups increased significantly. The MCV in the QC groups increased by 0.03 fL, whereas the MCV decreased by 1.37 fL, 1.09 fL, and 0.92 fL in the HHE55%, HHE70%, and HHE85% groups (*P* < 0.01), respectively, with the change being significant. The PLT in the QC, HHE55%, HHE70%, and HHE85% groups increased by 1.50 × 10^9^/L, 14.75 × 10^9^/L, 34.50 × 10^9^/L, and 34.75 × 10^9^/L, respectively. The changes in the HHE70% and HHE85% (*P* < 0.01) were significant. The PCT in the QC, HHE55%, HHE70%, and HHE85% groups increased by 0.01%, 0.02%, 0.04%, and 0.04%, respectively. The changes in the QC (*P* < 0.05), HHE70% (*P* < 0.01), and HHE85% (*P* < 0.01) groups were significant. The WBC in the QC, HHE55%, HHE70%, and HHE85% groups increased by 0.51 × 10^9^/L, 0.08 × 10^9^/L, 0.50 × 10^9^/L, and 0.91 × 10^9^/L, respectively. Changes in the HHE85% group were significant (*P* < 0.01). The LY in the QC, HHE55%, HHE70%, and HHE85% groups increased by 0.52 × 10^9^/L, 0.23 × 10^9^/L, 0.43 × 10^9^/L, and 0.04 × 10^9^/L, respectively. The change in the HHE70% group was significant (*P* < 0.05). The NE values in the QC and HHE55% groups decreased by 0.10 × 10^9^/L and 0.21 × 10^9^/L, respectively, whereas those in the HHE70% and HHE85% groups increased by 0.01 × 10^9^/L and 0.92 × 10^9^/L, respectively. Only the change in the HHE85% group (*P* < 0.01) was significant. The MO, EO, and BA values in the QC, HHE55%, HHE70%, and HHE85% groups showed no significant change (*P* > 0.05). These data are presented in Table 3.

**Table 3:** Values of blood cells pre- and post-exercise.

	Intervention	QC (*N* = 8)	HHE55% (*N* = 8)	HHE70% (*N* = 8)	HHE85% (*N* = 8)
RBC, 10^12^/L	Pre-Ex	5.06 ± 0.31	5.44 ± 0.62	4.86 ± 0.35	5.03 ± 0.34
	Post-Ex	5.10 ± 0.28	5.60 ± 0.63	5.11 ± 0.39^b^	5.17 ± 0.32^b^
HGB, g/L	Pre-Ex	151.25 ± 10.79	150.75 ± 14.22	138.50 ± 10.64	146.00 ± 8.60
	Post-Ex	150.38 ± 9.12	156.00 ± 17.76	145.63 ± 11.61^b^^,^ ^d^	149.50 ± 10.86^a^^,^ ^c^
HCT, %	Pre-Ex	45.10 ± 2.40	45.71 ± 4.00	42.73 ± 2.58	44.90 ± 2.18
	Post-Ex	45.46 ± 2.11	46.33 ± 4.23	44.40 ± 2.81^b^	45.70 ± 2.17^a^
MCV, fL	Pre-Ex	89.20 ± 2.53	84.93 ± 11.32	88.44 ± 9.16	89.36 ± 2.84
	Post-Ex	89.23 ± 2.48	83.56 ± 11.13^b^^,^ ^d^	87.35 ± 9.14^b^^,^ ^d^	88.44 ± 2.82^b^^,^ ^c^
PLT, 10^9^/L	Pre-Ex	215.88 ± 53.09	254.75 ± 70.22	217.13 ± 39.84	232.13 ± 36.59
	Post-Ex	217.38 ± 54.67	269.50 ± 56.69	251.63 ± 51.62^b^^,^ ^d^	266.88 ± 45.39^b^^,^ ^d^
PCT, %	Pre-Ex	0.24 ± 0.046	0.28 ± 0.075	0.25 ± 0.046	0.25 ± 0.033
	Post-Ex	0.25 ± 0.053^a^	0.30 ± 0.049	0.29 ± 0.056^b^^,^ ^c^^,^ ^e^	0.29 ± 0.039^b^^,^ ^d^^,^ ^e^
WBC, 10^9^/L	Pre-Ex	8.43 ± 3.57	9.03 ± 1.31	8.15 ± 2.20	6.21 ± 1.22
	Post-Ex	8.94 ± 3.04	9.11 ± 1.62	8.65 ± 1.95	7.12 ± 1.38^b^
LY, 10^9^/L	Pre-Ex	1.84 ± 0.96	2.31 ± 0.43	2.21 ± 0.38	2.41 ± 0.68
	Post-Ex	2.36 ± 0.44	2.54 ± 0.53	2.64 ± 0.44^a^	2.45 ± 0.62
NE, 10^9^/L	Pre-Ex	6.09 ± 3.60	6.10 ± 1.34	5.20 ± 1.99	3.16 ± 0.84
	Post-Ex	5.99 ± 3.15	5.89 ± 1.39	5.21 ± 1.59	4.08 ± 1.35^b^
MO, 10^9^/L	Pre-Ex	0.43 ± 0.191	0.54 ± 0.106	0.56 ± 0.130	0.46 ± 0.092
	Post-Ex	0.49 ± 0.173	0.59 ± 0.164	0.61 ± 0.173	0.48 ± 0.089
EO, 10^9^/L	Pre-Ex	0.09 ± 0.079	0.09 ± 0.053	0.17 ± 0.160	0.10 ± 0.058
	Post-Ex	0.09 ± 0.069	0.08 ± 0.060	0.18 ± 0.162	0.10 ± 0.068
BA, 10^9^/L	Pre-Ex	0.02 ± 0.011	0.02 ± 0.013	0.03 ± 0.019	0.02 ± 0.009
	Post-Ex	0.03 ± 0.021	0.03 ± 0.014	0.03 ± 0.019	0.03 ± 0.017

**Notes:**

Data are presented as mean ± standard deviation (SD).

Abbreviations: Pre-Ex, pre-exercise; Post-Ex, post-exercis; WBC, white blood cell count; RBC, red blood cell count; HGB, hemoglobin; PLT, platelets count; HCT, hematocrit; MCV, mean red blood cell volume; PCT, plateletcrit; MPV, mean platelet volume; LY, lymphocyte count; NE, neutrophil granulocyte; MO, number of monocytes; EO, number of eosinophils; BA, number of basophils.

a *P* < 0.05.

b *P* < 0.01, compared with pre-exercise vakyes.

c *P* < 0.05.

d *P* < 0.01, compared with QC.

e *P* < 0.05, compared with HHE55%.

### Serum IL-3, EPO, and sTfR

**Figure 1:**
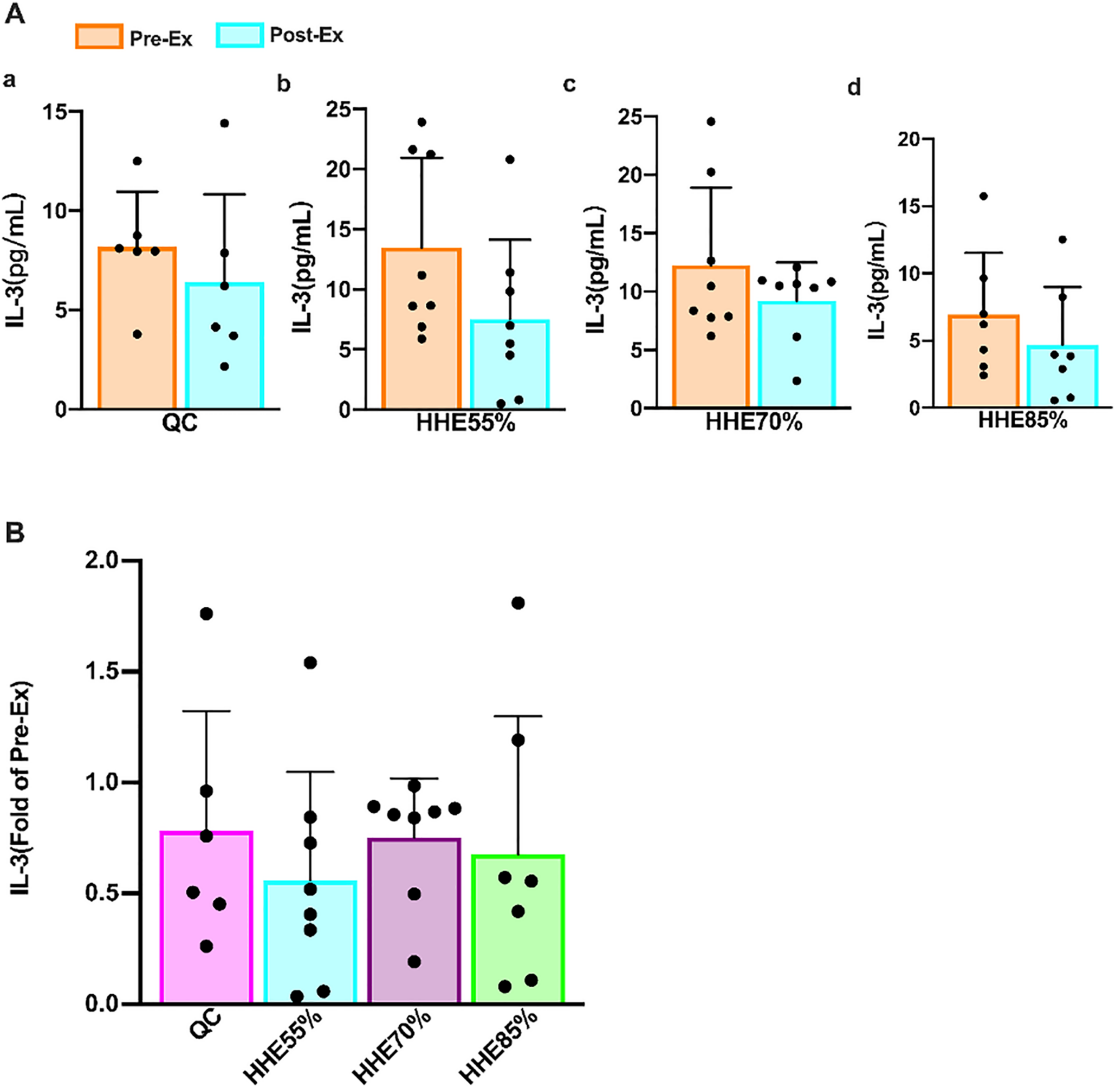
Effects of acute exercise on human serum IL-3 levels. (A) The IL-3 level in the QC, HHE55%, HHE70%, and HHE85% groups pre- and post-exercise. (B) The fold of IL-3 value in the QC, HHE55%, HHE70%, and HHE85% groups post-exercise compared with that pre-exercise. Abbreviations: Pre-Ex, pre-exercise; Post-Ex, post-exercise; IL-3, interleukin 3; QC, quiet control bout; HHE55%, 55% VO_2_max intensity exercise bout; HHE70%, 70% VO_2_max intensity exercise bout; HHE85%, 85% VO_2_max intensity exercise bout.

The results of the paired sample *t*-test showed that the IL-3 levels in all groups decreased compared with the pre-exercise values, but no significant difference was observed (*P* > 0.05) (Fig. 1A). The results of one-way ANOVA showed that the IL-3 levels were not affected by the exercise intensity. Bonferroni’s *post hoc* comparison showed that there were no significant differences between any pair of groups (*P* > 0.05) (Fig. 1B).

**Figure 2:**
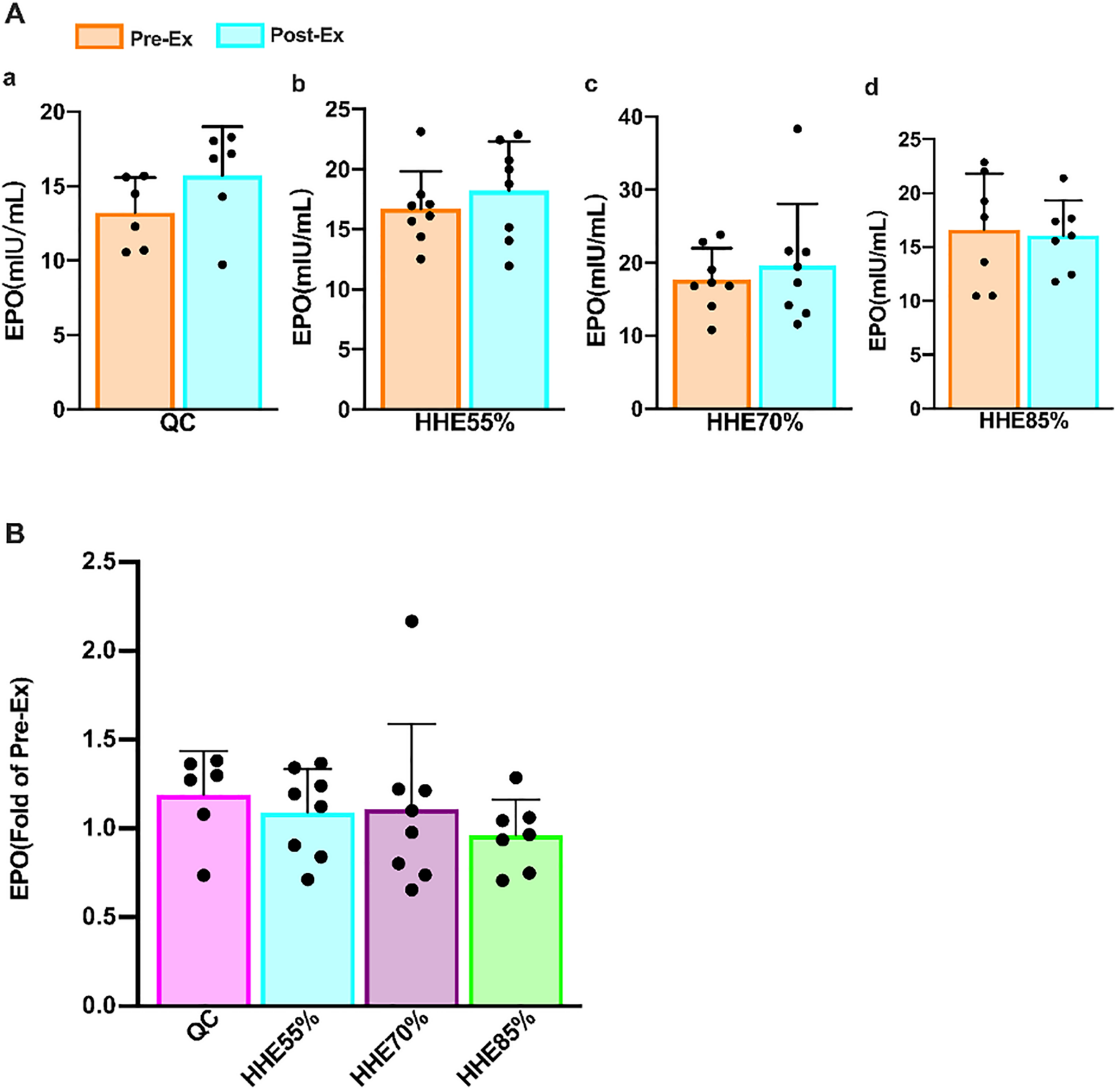
Effects of acute exercise on human serum EPO levels. (A) The EPO levels in the QC, HHE55%, HHE70%, and HHE85% groups pre- and post-exercise. (B) The fold of EPO level in the QC, HHE55%, HHE70%, and HHE85% groups post-exercise compared with that pre-exercise. Abbreviations: Pre-Ex, pre-exercise; Post-Ex, post-exercise; EPO, erythropoietin; QC, quiet control bout; HHE55%, 55% VO_2_max intensity exercise bout; HHE70%, 70% VO_2_max intensity exercise bout; HHE85%, 85% VO_2_max intensity exercise bout.

The results of the paired sample *t*-test showed that the EPO in the QC, HHE55%, and HHE70% groups increased compared with the pre-exercise values, whereas that in the HHE85% decreased marginally; however, there were no significant differences (*P* > 0.05) (Fig. 2A). One-way ANOVA results showed that EPO was not affected by exercise intensity. Bonferroni’s *post hoc* comparison showed that there were no significant differences between the values in any pair of groups (*P* > 0.05) (Fig. 2B).

**Figure 3:**
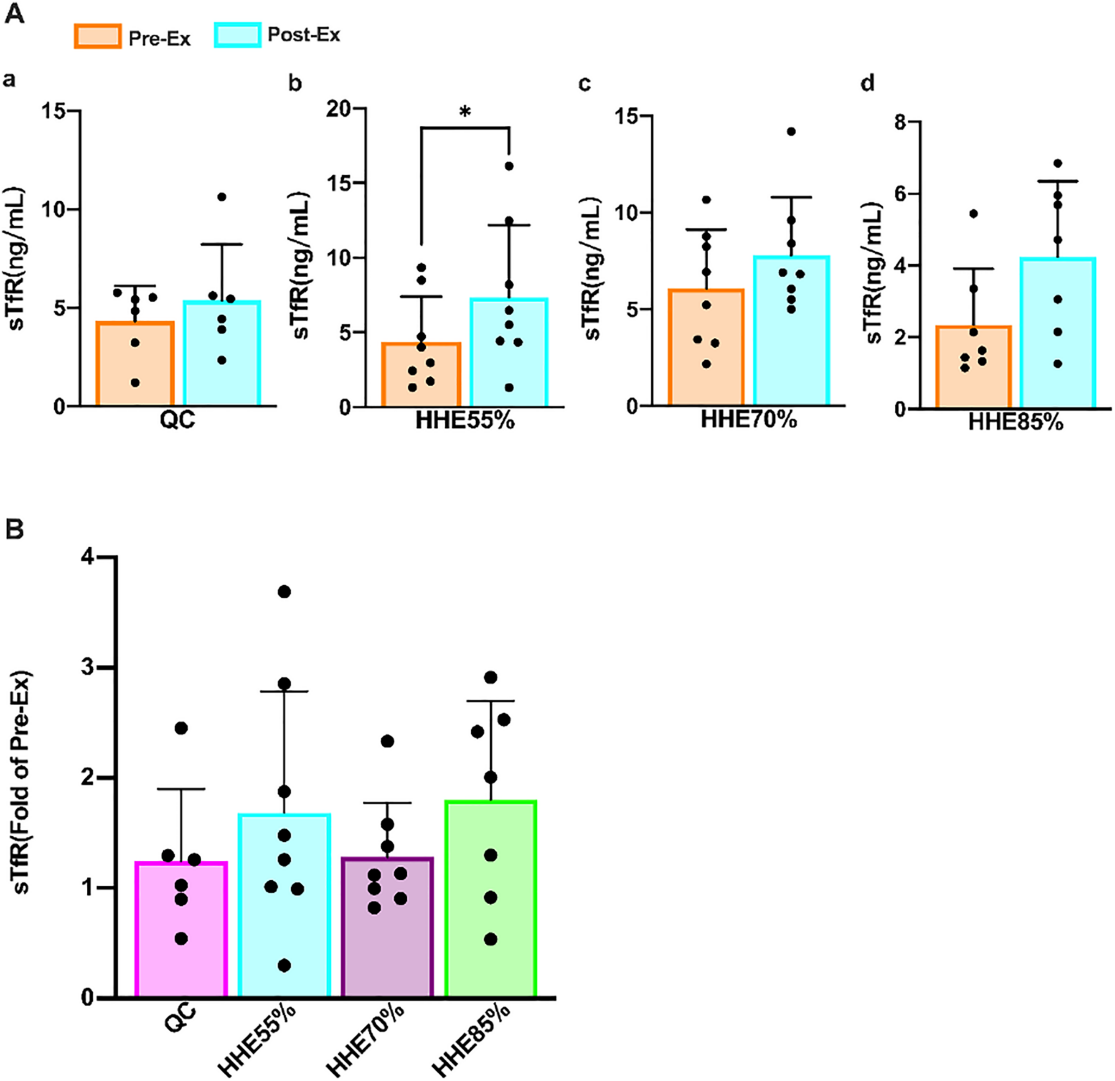
Effects of acute exercise on human serum sTfR levels. (A) The sTfR levels in the QC, HHE55%, HHE70%, and HHE85% groups pre- and post-exercise. (B) The fold of sTfR level in the QC, HHE55%, HHE70%, and HHE85% groups post-exercise and pre-exercise. Abbreviations: Pre-Ex, pre-exercise; Post-Ex, post-exercise; sTfR, soluble transferrin receptor; QC, quiet control bout; HHE55%, 55% VO_2_max intensity exercise bout; HHE70%, 70% VO_2_max intensity exercise bout; HHE85%, 85% VO_2_max intensity exercise bout.

Results of the paired sample *t*-test showed that the sTfR levels in all groups increased from the pre-exercise levels, and the level in the HHE55% group increased significantly (*P* < 0.05) (Fig. 3A). One-way ANOVA showed that sTfR was not affected by exercise intensity. Bonferroni’s *post hoc* comparison showed that there were no significant differences between any pair of groups (*P* > 0.05) (Fig. 3B).

## Discussion

### Effects of SBOE on NBM, ST, SaO_2_, and PI

Exercise in hot and/or humid environments can lead to exertional heat stress and exertional rhabdomyolysis and reduce perception and athletic ability owing to endogenous heat production and exogenous load, which directly reduces the quality of work, exercise training, and exercise performance (Skwarczynski et al., 2010; Melikov & Kaczmarczyk, 2012; Guy et al., 2015; Periard, Racinais & Sawka, 2015; Willmott et al., 2019; McCubbin et al., 2020; Carneiro et al., 2021). Prolonged heat stress can disrupt the thermoregulatory, cardiovascular, and gastrointestinal systems, leading to severe concerns for an athlete’s health and performance (McCubbin et al., 2020). Some major sporting events are held in hot and/or humid conditions, which can be challenging for several athletes (Gerrett et al., 2019). Nutritional strategies before, during, and after exercise and heat acclimation are suitable methods to alleviate exercise heat stress (McCubbin et al., 2020; Sawka et al., 2011). A study showed that the body mass of participants decreased significantly after the first day of acute exercise-heat stress (Willmott et al., 2019). Our results showed that the NBM in all exercise groups decreased significantly from the pre-exercise values and showed significant difference with the values in the QC group, similar to the findings reported by Willmott et al. (2019).

Body temperature is affected considerably by our environment and the work we do, and body surface temperature (Periard, Racinais & Sawka, 2015), sublingual temperature (Singh & Kumar, 2019), forehead temperature (Dzien et al., 2021), ear canal temperature (Mueller et al., 2012), and rectal body temperature (Meade et al., 2020; Levy, Allender & Keller, 2020) are commonly used as detection indices (van der Vinne et al., 2020). Prolonged exercise increases skin, muscle, and core body temperatures (Ely et al., 2007). A study of medical technician students during simulated work activities in a hot environment showed that the core temperature, skin temperature, and mean body temperature of participants were higher under heated conditions than under neutral conditions (Gerhart et al., 2020). Our results showed that the ST was unaffected by the exercise intensity in an HHE. The post-exercise ST in all groups was significantly greater than the pre-exercise ST, and the HHE55% group showed the least increase, indicating that low-intensity exercise may be more conducive to controlling the rise of body temperature in an HHE.

The SaO_2_ indicates the physiological oxygen-carrying capacity. An individual’s body temperature increases by 1 °C, whereas their blood oxygen saturation decreases by 0.27% (Qiu et al., 2020). Our findings showed that SaO_2_ was unaffected by exercise intensity, whereas PI was affected significantly by the exercise intensity in an HHE. The PI levels in all exercise groups were significantly different from that in the QC group, but there was no significant differences among the values in any pair of exercise groups. The post-exercise SaO_2_ in all exercise groups had decreased compared with the corresponding pre-exercise values, but only the change in the HHE55% group was significant, indicating that blood oxygen utilization is optimum at this exercise intensity. The post-exercise PI in all exercise groups was significantly higher than the pre-exercise PI, and the values were significantly different in all exercise groups compared to that in the QC group, suggesting that the choice of low- and moderate-intensity exercise may be more appropriate in an HHE.

### Effects of SBOE on blood cells

The human body is more prone to dehydration, mineral loss, and weight loss in an HHE (Adams et al., 2018; James et al., 2017). Dehydration may also reduce cardiac filling and affect the body’s ability to regulate blood pressure (Stoehr et al., 2011). Research showed that plasma volume decreases significantly during heat exposure and after exercise at a certain level of dehydration, whereas it is better maintained during and after exercise (Jimenez et al., 1999). Exercise can lead to acute and chronic increases in the HCT (Pichon, Connes & Robach, 2016). Meanwhile, the HGB and HCT values decrease in athletes after single and repeated bouts of exercise (Alberty et al., 2021). Moreover, physical exercise research until exhaustion showed that erythrocytes do not undergo alterations (Siquier-Coll et al., 2019). Boukelia, Gomes & Florida-James (2018) used Bonferroni-adjusted *post hoc* tests to show that PLT is significantly affected by exercise and environmental conditions at two different times of day (09:00 hrs and 18:00 hrs). Our results showed that HGB, MCV, PLT, and PCT were affected significantly by exercise intensity, whereas others were not. The HGB, MCV, PLT, and PCT values in the HHE70% and HHE85% groups changed significantly compared with those in the QC group. The PCT values of the HHE70% and HHE85% groups were significantly different from those in the HHE55% group. The RBC, HGB, HCT, MCV, PLT, and PCT values in the HHE70% and HHE85% groups changed significantly compared with the pre-exercise values, whereas only the MCV changed significantly in the HHE55% group. We speculated that the decrease in MCV may be associated with exercise, which severely affected the RBC. Moderate exercise can strengthen the immunity of athletes, but the high intensity of long-term exercise temporarily weakens the immunity of athletes (Simpson et al., 2006). Exposure to heat may mobilize more white blood cells into the circulation to increase physiological demands (Mitchell et al., 2002). A study on highly trained runners who performed a 10 km time trial run in an HHE showed that the posttrial WBC, NE, LY, and MO increased significantly compared with the pretrial values (Boukelia, Gomes & Florida-James, 2018). Similarly, completing a 100-mile recreational cycling race in an HHE resulted in a significant increase in the total number of circulating white blood cells (Luk et al., 2016). We found that the WBC, LY, NE, MO, EO, and BA were not affected by exercise intensity during SBOE in an HHE. The WBC and NE in the HHE85% group and LY in the HHE70% group changed significantly compared with the pre-exercise values, whereas the other values did not change. This indicates that low-intensity exercise may be better for SBOE in an HHE, because low-intensity exercise may have a negligible effect on the blood.

### Effects of SBOE on serum IL-3, EPO, and sTfR

IL-3 produced by monocytes and lymphocytes, also known as multiple colony-stimulating factors, indicates physiological hematopoietic function. Granulocyte-macrophage colony-stimulating factor (GM-CSF) plays a crucial role in bone marrow differentiation (de Rezende et al., 2020). To date, studies on IL-3 were primarily confined to hematopoietic, antitumor, and immunomodulatory effects (Borriello et al., 2019). Nevertheless, with more research, IL-3 has been found to play an essential role in diseases associated with inflammation (Hu et al., 2019). Although studies have shown that chronic exercise can boost immunity (Sellami et al., 2018), whereas acute exercise may reduce immunity (Kakanis et al., 2010; Campbell & Turner, 2018), findings from a recent study were contradictory (Spielmann et al., 2016). An early study showed that plasma IL-3 concentration is unaltered with training and/or with exercise (Mucci et al., 2000). We found that the IL-3 levels are unaffected by acute exercise and exercise intensity, consistent with the findings by Mucci et al. (2000). IL-3 levels decreased after an SBOE in an HHE, but no significant difference was observed between any pair of exercise groups. After an SBOE, the WBC and NE of the HHE85% group increased significantly. These results indicate that high-intensity exercise can increase the stimulation of the body, leading to acute infection or inflammation.

EPO acts as the primary regulator of red blood cell maturation. It is primarily synthesized in the kidney and to a lesser extent in the liver and brain (Sgro et al., 2018; Jelkmann, 2011). Its functions include promoting the proliferation and differentiation of erythrocyte progenitor cells, maintaining the number of erythrocytes and hemoglobin (Jelkmann, 2011), mediating apoptosis (Kumral et al., 2006), promoting axonal bud formation (Garg, Sharma & Bansal, 2018), facilitating immune-modulatory effects (Nairz et al., 2012), and improving heart function (Zhang et al., 2016). EPO was shown to help the Hb concentration remain relatively constant and accelerate the recovery of red blood cells after blood loss. Healthy individuals require limited amounts of EPO to maintain a stable state (Jelkmann, 2011). EPO is also used to treat various diseases, although it may exert adverse effects in patients with anemia and chronic heart failure, such as elevated blood pressure, thrombosis, and seizures (Rao, Binbrek & Sobel, 2008). A recent clinical study has shown that EPO is still relatively safe to treat chronic heart failure (Zhang et al., 2016). Recombinant human erythropoietin also has multiple functions, such as reducing the expression of inflammatory factors TNF-α and IL-1β after acute injury, improving motor function, reducing inflammation, and facilitating neuroprotection and functional recovery (Zhou et al., 2017). EPO generation depends on the partial pressure of tissue oxygen, and EPO expression is also activated when the arterial oxygen partial pressure decreases or O_2_ affinity increases in the blood (Jelkmann, 2011). Under normal circumstances, a healthy serum EPO concentration is 6-32 IU/L, but significant differences are observed among individuals (Jelkmann, 2011; Eckardt & Kurtz, 2005). Heat stress resulting from endurance exercise in hypoxia (heat and hypoxic conditions) did not augment the EPO response (Yatsutani et al., 2019). Our data showed that EPO levels are unaffected by an acute exercise bout and exercise intensity. There was no significant difference between the values observed in different bouts and the pre-exercise values, consistent with the research findings of Yatsutani et al. (2019). The EPO level in the HHE85% group decreased, whereas it increased in the other groups, indicating that high-intensity exercise in an HHE may decrease the EPO level, whereas low- and moderate-intensity may increase it.

With the improvement of our quality of life and understanding of science, people are becoming increasingly aware of the importance of exercise or physical activity. It is essential to pay attention to nutritional supplements and rest and take an appropriate approach to exercise. Young people may have a high demand for iron bioavailability owing to rapid growth and sports participation (Shoemaker et al., 2019). Some athletes may even have unsatisfactory iron reserves throughout the season (Smith et al., 2020), and iron overload can increase EPO resistance (Zhang, 2020). sTfR, red blood cell-free protoporphyrin, transferrin saturation (TS), serum iron (SI), and total iron-binding capacity play a significant clinical role in the diagnosis and differential diagnosis of abnormal iron metabolism (Yang, Yang & Liang, 2013). Physical exercise reduces the SI and TS and increases sTfR levels (Di Santolo et al., 2008), and sTfR levels exhibit a moderate correlation with the athletic performance of young female athletes, which may reflect the increase in their red blood cell production rate during their growth spurt (Di Santolo et al., 2008; Shoemaker et al., 2020). Simultaneously, the relationship between hemoglobin levels and the performance of young male athletes was also relatively strong (Shoemaker et al., 2019). In addition, sTfR is a sensitive marker for erythropoiesis stimulation (Brugnara, 2003). Our findings showed the sTfR is unaffected by exercise intensity. After an SBOE in an HHE, the sTfR levels in all groups increased, and that in the HHE55% group increased significantly.

## Conclusion

In conclusion, low-intensity exercise improves oxygen utilization, leads to a lower rise in the body temperature and a greater rise in the PI, exerts minimal effect on blood cells, and causes a significant increase in the sTfR levels. Low- and moderate-intensity SBOE can increase the serum EPO and sTfR levels and decrease the serum IL-3 level in an HHE. A high-intensity load could increase the risk of inflammation. Low-intensity exercise may be more appropriate for an SBOE in an HHE.

However, despite the strength of our findings, we acknowledge that there are multiple limitations in this study. First, in the laboratory setting of the study, participants were exposed to an HHE for only a limited period. Second, owing to complex and interrelated interactions, we cannot confirm the exact mechanisms underlying our findings. Third, we tested only a limited number of factors that promote hematopoiesis and only immediately after SBOE. In future studies, we can conduct continuous follow-up tests after exercise to understand how each indicator changes. In addition, we must also test the factors that inhibit hematopoiesis.

## Supplemental Information

10.7717/peerj.18603/supp-1 Supplemental Information 1 The baseline participant characteristics.

10.7717/peerj.18603/supp-2 Supplemental Information 2 Body weight, Sublingual temperature, SaO_2_, and PI.

10.7717/peerj.18603/supp-3 Supplemental Information 3 Data of participants’ blood cells.

10.7717/peerj.18603/supp-4 Supplemental Information 4 Data of EPO, sTfR, and IL-3.

## Additional Information and Declarations

### Data Availability

The following information was supplied regarding data availability: The raw measurements are available in the Supplemental Files.
